# Treatment of oral cancers during pregnancy: a case-based discussion

**DOI:** 10.1186/s40463-019-0331-1

**Published:** 2019-02-04

**Authors:** Kiyoshi Sato, Hiroaki Shimamoto, Yumi Mochizuki, Hideaki Hirai, Hirofumi Tomioka, Risa Shimizu, Eriko Marukawa, Haruhisa Fukayama, Ryoichi Yoshimura, Hiroo Ishida, Hiroyuki Harada

**Affiliations:** 1 Department of Oral and Maxillofacial Surgery, Graduate School of Medical and Dental Sciences, Tokyo Medical and Dental University, 1-5-45 Yushima, Bunkyo-ku, Tokyo, 113-8549 Japan; 2 Department of Anesthesiology and Clinical Physiology, Graduate School of Medical and Dental Sciences, Tokyo Medical and Dental University, Tokyo, Japan; 3 Department of Radiation Therapeutics and Oncology, Graduate School of Medical and Dental Sciences, Tokyo Medical and Dental University, Tokyo, Japan; 4 Department of Medical Oncology, Showa University School of Medicine, Tokyo, Japan

**Keywords:** Oral cancer, Pregnancy, Surgical therapy, Chemotherapy, Radiotherapy

## Abstract

**Background:**

Malignancies occur in approximately 1:1000 pregnancies; the most common being breast (46%) and hematological (18%) malignancies. Oral cancers account for only 2% of all cancers in pregnant women, and there are no standard guidelines for the treatment of oral cancer during pregnancy.

**Methods:**

Between 2007 and 2014, our department managed 1109 patients with oral cancers; four (0.4%) had tongue carcinomas during pregnancy. These cases were retrospectively reviewed.

**Results:**

The four women were aged 29–39 (median 32.5) years. Two underwent partial glossectomy at 39 and 40 weeks’ gestation, respectively, one received radiotherapy at 17 weeks’ gestation, and one underwent supraomohyoid neck dissection and hemi-glossectomy with a forearm flap reconstruction.

**Conclusion:**

In addition to tumor factors, the wishes of the patient and her family, gestational age, and fetal and maternal conditions are important factors in deciding on a treatment protocol. Moreover, treatment decisions require multidisciplinary approach.

## Introduction

When malignant tumors are detected during pregnancy, the prognosis of the mother and/or the fetus may be compromised, depending on the treatment method used. Moreover, it is often difficult to decide on an appropriate treatment policy. It is reported that malignant tumors occur in 1:1000 pregnancies [1, 2]. However, because maternal age of pregnancy is increasing and the age at which cancers occur is decreasing, it is likely that the incidence of malignancy during pregnancy will increase. Regarding the treatment of such cancers, it is necessary to provide sufficient information, after fully collaborating with other departments such as obstetrics and gynecology, personalized care in accordance with the wishes of the patient and her family. Understanding the particular characteristics of a tumor during pregnancy is crucial. However, there are few reports on the management of oral cancers during pregnancy. The aim of this study is a retrospective case review from single institution experience.

## Material and methods

A total of 1109 patients with oral cancer were managed at our department of Oral and Maxillofacial Surgery at the Tokyo Medical and Dental University between 2007 and 2014. Of these, four (0.4%) were pregnant women with a pathologic diagnosis of squamous cell carcinoma (SCC) of the tongue. The clinicopathologic data of these patients—including age and gestational age at diagnosis, TNM classification, adverse events, smoking, and alcohol use—were reviewed and the treatment period, type of treatment received, and fetal and maternal outcome were evaluated.

Ethical approval was granted by the institutional review board at the Dental Hospital of the Tokyo Medical and Dental University (No. D2015–600).

## Results

Table 1 shows the patients’ characteristics, treatment received, and maternal and fetal outcomes. The median age at diagnosis was 32.5 years (range: 29–39 years). Two patients had stage T2N0M0, one had stage rT1N0M0, and one had stage rT0N0M1 disease. Two patients had been smokers before pregnancy, the others were non-smokers. None consumed alcohol. Two patients underwent surgery during pregnancy (at 39 and 40 weeks’ gestation, respectively), one was treated with radiotherapy during pregnancy (at 17 weeks’ gestation), and the last patient underwent surgery after terminating the pregnancy. Each case is presented in detail below. Given the retrospective nature of this study, HPV status was not available.

**Table 1:** Patients’ characteristics and treatment, and maternal and fetal outcomes

	Age,years	TNM classification	Smoking	Alcohol	Age of pregnancy at diagnosis,weeks	Treatment period, weeks	Treatment during pregnancy	Fetal outcome	Maternal outcome
Case 1	29	T2N0M0	Previous smoker (before pregnancy)	No	16	–	–	Pregnancy terminated	Alive with no evidence of disease
Case 2	33	T2N0M0	Previous smoker (before pregnancy)	No	8	13	partial glossectomy	Delivered a healthy baby at 39 weeks	Alive with no evidence of disease
Case 3	32	rT1N0M0	non-smoker	No	22	25	partial glossectomy	Delivered a healthy baby at 40 weeks	Alive with no evidence of disease
Case 4	39	rT0N0M1 (lung)	non-smoker	No	5	17	radiotherapy	Cesarean section performed at 29 weeks	Died

### Case 1

In February 2007, a 30-year-old woman at 16 weeks’ gestation was referred to our department with a one-month history of tongue pain. The patient was an ex-smoker, but had no history of alcohol consumption. A hard, endophytic tumor was present in the midsection of the tongue on the right. The lesion measured 2.6 × 2.2 × 0.8 cm and extended to the floor of the mouth (cT2N0M0).

Neither the patient nor her family wished to continue this pregnancy, preferring to concentrate on treating the SCC. Two weeks after terminating the pregnancy, the patient underwent a supraomohyoid neck dissection and hemi-glossectomy with reconstruction using a free forearm flap. Her post-operative course was uneventful. Pathologic examination of the resected specimen confirmed a well-differentiated SCC with clear margins and no cervical lymph node metastases; it was classified as a pT2 N0 tumor. In the 11.5 years since undergoing surgical treatment for this tumor, the patient has remained healthy, with no recurrence.

### Case 2

**Fig. 1:**
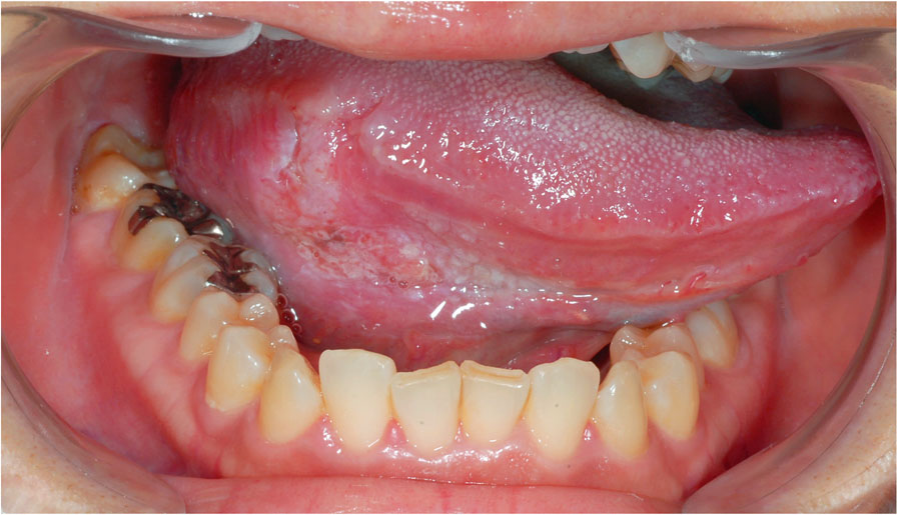
Preoperative oral findings of case 2. The superficial ulcerarive and painful tumor existed over the right border of the tongue with 2.3 × 1.6 cm

In November 2010, a 33-year-old woman who was 8 weeks’ pregnant was referred to our department with a two-month history of tongue pain. The patient was an ex-smoker but had no history of alcohol consumption. Physical examination revealed a painful, ulcerated lesion measuring 2.3 × 1.6 × 4.1 cm on the right side of the tongue (cT2N0M0) (Fig. 1). After consulting with an obstetrician, it was decided to avoid performing contrast-enhanced computed tomography (CT) and to use β-lactam antibiotics and acetaminophen in the patient’s perioperative management.

At 13 weeks’ gestation, the patient underwent a trans-oral partial glossectomy under general anesthesia. Fentanyl citrate, thiamylal sodium, and sevoflurane were used for anesthetic induction, and sevoflurane and oxygen in air were used for maintenance of anesthesia. Lidocaine 1% with adrenaline (epinephrine) 1:300,000 was used for local anesthesia. Cefazolin was used to prevent postoperative infection, and acetaminophen was used for analgesia. During surgery, the fetus was monitored by ultrasonic examination. The operation time was 1 h 7 min and the anesthesia time was 2 h 1 min. The patient’s postoperative course was uneventful. The tumor margin was positive, therefore, the patient underwent trans-oral wide excision of the lesion under local anesthesia at 17 weeks’ gestation. The margins of the resected specimen were clear. The child was delivered at term and developed normally. In the 7 years since the operation, the patient has remained free of the disease.

### Case 3

In March 2012, a 32-year-old woman developed SCC recurrence of the right side of her tongue. At that time, the lesion measured 0.7 × 0.4 cm (rT1N0M0). She was then 22 weeks’ pregnant. She was a non-smoker and had no history of alcohol consumption. In consultation with the doctor in charge of obstetrics and gynecology, it was decided that the following drugs be used in the patient’s perioperative management: cefazolin or cefcapene pivoxil hydrochloride (antibiotics) and flurbiprofen axetil and diclofenac sodium (analgesics).

At 25 weeks of pregnancy, a trans-oral partial glossectomy was performed under general anesthesia. Pathologic examination confirmed a well-differentiated SCC with clear margins. Remifentanil hydrochloride, thiamylal sodium, and sevoflurane were used for anesthetic induction, and remifentanil hydrochloride and oxygen in air were used for maintenance of general anesthesia. Lidocaine 1% with adrenaline (epinephrine) 1:300,000 was used for local anesthesia. Cefazolin and cefcapene pivoxil hydrochloride were used to prevent postoperative infection, and acetaminophen was used for analgesia. Ultrasonic examination was used to monitor the fetus intraoperatively. The operation time was 1 h 13 min and the anesthesia time was 2 h 22 min. The patient’s postoperative course was uneventful. Four months after the operation, she delivered a healthy baby. Six years after the final operation, the patient remains free of the disease.

### Case 4

**Fig. 2:**
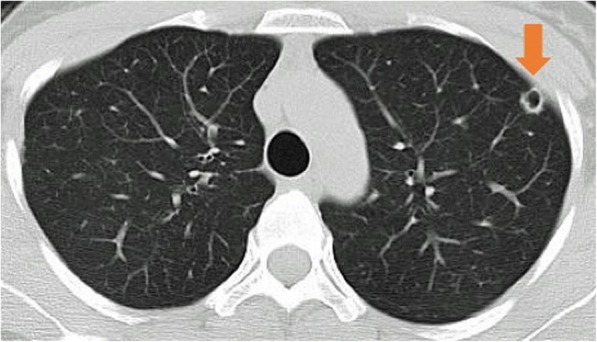
Metastasis findings of case 4. CT image shows multiple pulmonary metastases

In December 2011, a 38-year old woman was diagnosed with T2 N0 SCC of the left side of the tongue; the diagnosis was confirmed by pathologic examination of a biopsy specimen. Brachytherapy was delivered via a Cs needle, up to a dose of 70 Gy. In May 2012, the patient developed cervical lymph node metastasis and underwent a left modified radical neck dissection. Cervical lymph node metastases were found in two (out of 48) level II lymph nodes, with extracapsular spread. In December 2012, CT revealed multiple pulmonary metastases, as shown in Fig. 2. However, the patient was at this point five-weeks’ pregnant. She and her family wished to continue the pregnancy, and therefore rejected the option of chemotherapy. Radiotherapy was thus administered for the pulmonary metastases. The patient underwent a caesarean section at 29 weeks’ gestation. Thereafter, she received 6 cycles of chemotherapy with cisplatin and 5-fluorouracil. The SCC relapsed in the patient’s neck and lungs, and she died 10 months after undergoing chemoradiotherapy.

## Discussion

The probability of having a malignant tumor during pregnancy is approximately 0.1%, and approximately 2% of all malignancies in pregnancy are oral cancers [3]. This low incidence is thought to be because oral cancers are less common in young people and occur more commonly in men [4]. Over a seven-year period in our department, only four (0.4%) oral cancers occurred in pregnant women; their median age was 32.5 years. However, it is possible that the incidence of oral cancers during pregnancy may increase because of the social trend toward later child-bearing. A review of the literature of oral cancer during pregnancy is presented in Table 2 [3–22]. We found 39 cases of oral cancer during pregnancy in 38 patients, including the present patient. These articles were identified during PUBMED search, with terms “pregnancy”, “oral cancer” and “tongue cancer”. There were no exclusion criteria. Thirty-seven cases (97.4%) with a diagnosis of tongue cancer were identified by means of a review.

**Table 2:** Characteristics of 39 pregnancy in 38 oral cancer patients

Characteristics		
Age, year (average)	18–40	(30.9)
Age of pregnancy at diagnosis, weeks (average)		
	5–31	(18.9)
Primary Site		
Tongue	37	(97.4%)
Hard plate	1	(2.6%)
Stage		
I	8	(20.5%)
II	10	(25.6%)
III	4	(10.3%)
IV	14	(35.9%)
NA	3	(7.7%)
Pathological diagnosis		
SCC	36	(94.7%)
Others	2	(5.3%)
Smoking		
Yes	3	(7.9%)
No	20	(52.6%)
Before pregnancy	6	(15.8%)
NA	9	(23.7%)
Alcohol		
Yes	0	(0%)
No	29	(76.3%)
NA	9	(23.7%)
Treatment during pregnancy		
Surgery	22	(56.4%)
CT	5	(12.8%)
RT	11	(28.2%)
None	9	(23.1%)
Fetal outcome		
Delibery	28	(73.7%)
Cesarean	7	(18.4%)
Termination	2	(5.3%)
Spontaneous abortion	1	(2.6%)
Maternal outcome		
NED	25	(65.8%)
Died	10	(26.3%)
NA	3	(7.9%)

*SCC* squamous cell carcinoma, *NA* not available, *CT* chemotherapy, *RT* radiotherapy, *NED* not evidence disease

### General considerations

The physiologic changes that occur during pregnancy may influence malignancy in various ways. For example, there are many immunosuppressive factors in the serum of pregnant women, particularly related to inhibition of cellular immunity, such as CD8+ T-cells; this results in an increase in antitumor effector cells as pregnancy progresses [23]. Conversely, the number of CD4+ T-cells involved in humoral immunity decreases throughout pregnancy. The Th1/Th2 balance is significantly lower in the pregnant than in the non-pregnant state. During the second and third trimesters of pregnancy, antitumor activity (via Th1) is suppressed while tumor growth (via Th2) is promoted [24], and the number and function of natural killer cells decreases significantly [23, 25]. Furthermore, cancer proliferation has been reported in patients with AIDS and those receiving potent immunosuppressive therapy [26]. These findings highlight a relationship between immune function and the development of cancer. However, it is thought that the effect of declining anti-tumor immunity in pregnancy is mild and does not affect prognosis [27]. In case 3, surgery was deferred from the eighth to the thirteenth weeks of pregnancy. During that time the tumor progressed, but no particularly rapid progression or metastasis was observed.

In terms of imaging studies, CT is said not to cause adverse effects such as fetal malformation or death when the exposure is ≤50 mGy. Moreover, fetal absorbed doses of < 100 mGy are said to be no reason for terminating a pregnancy [28, 29]. Magnetic resonance imaging (MRI) is thought to be safe to perform throughout pregnancy; there is no significant difference in the incidence of stillbirths, fetal malformations, developmental abnormalities, and growth abnormalities between children exposed to MRI in-utero and those not exposed [30]. However, gadolinium contrast medium is reported to be associated with an increased incidence of stillbirth and of rheumatic symptoms and inflammatory skin diseases by 4 years of age in those exposed to it in utero; its use should be avoided in pregnancy [31]. For nuclear medicine investigations, the fetal exposure dose of 18 F-fludeoxyglucose used in positron emission tomography/ CT (PET/CT) is set at 20.9–23.2 mGy [32]. The exposure dose can be reduced by increasing maternal hydration and urination [32]. However, as radioisotopes stored in the bladder may affect the fetus, PET/CT was not performed for any of the patients whose cases have been presented. Simple MRI was performed at 12 weeks in case 2 and at 23 weeks in case 3, and ultrasonography was performed to examine cervical lymph nodes. CT and PET/CT examinations were not performed for any of the four patients.

There are no clear guidelines for the treatment of oral cancer during pregnancy. When deciding on a treatment strategy, the patient’s social background—such as family background, pregnancy history, gestation—as well as the type of malignant tumor and its stage, should be taken into consideration. In early pregnancy, it is possible to terminate the pregnancy so as to concentrate on treating the cancer. Because there are restrictions on terminating a pregnancy and inducing childbirth in mid-pregnancy, there is a high possibility at this stage of choosing treatment while continuing with the pregnancy. In the latter part of the pregnancy, a caesarean section may be performed after 30–34 weeks’ gestation, or delivery may be induced [18, 33]. According to the literature, treatment of oral cancer during pregnancy is diverse, such as surgery (56.4%), chemotherapy (12.8%), radiotherapy (28.2%), no treatment during pregnancy (23.1%) (Table 2).

### Surgical management

Regarding surgical therapy, it is necessary to be aware that because both cell-mediated and humoral immunity are suppressed during pregnancy, the risk of postoperative infection is high [3]. In the second trimester, the anatomic and physiologic changes in the mother’s body are relatively mild, making it the safest period in which to perform surgery [20, 34]. Commonly used anesthetic agents are reported not to carry an increased risk of teratogenicity. However, it is important to avoid the use of other teratogenic drugs (such as tetracycline antibiotics and phenobarbital) and to control maternal and fetal circulation and respiration intraoperatively. In pregnancy, the body is prone to hypoxemia because functional residual capacity decreases and oxygen consumption increases by approximately 20% [34–36]. Furthermore, since uteroplacental blood flow is not under autonomic regulation, it is important to maintain maternal blood pressure and oxygenation [35]. In cases 2 and 3, surgery was performed under general anesthesia at weeks 13 and 25, respectively; the subsequent delivery, and maternal and fetal growth were unaffected. Local anesthetics are administered cautiously to pregnant women in Japan as safety regarding their use in pregnancy has not been established. In terms of safety in pregnancy, the US Food and Drug Administration classifies lidocaine, etidocaine, and prilocaine as category B drugs and bupivacaine as a category C drug because all can cause fetal bradycardia. All local anesthetic agents should be administered in the lowest possible dose that provides pain relief [37].

### Radiation therapy conditions

Pregnancy is not an absolute contraindication to receiving radiation therapy. The threshold for abortion, fetal death, microcephaly, and fetal malformations is set at 100 mGy; the probability decreases at 50–100 mGy and doses of ≤50 mGy are considered safe [38, 39]. However, gestational age plays a role, as radiation may be lethal to the fetus during days 0–10 days, prior to implantation. This period—even up to 2–14 weeks—is considered a high-risk period for fetal malformations and developmental disorders. From 15 weeks’ gestation until the start of the third trimester, radiation therapy has the least adverse effect on the fetus, but radiotherapy delivered in the third trimester is associated with an increase in the incidence of pediatric cancers [39]. When administering radiotherapy, it is necessary to minimize exposure due to photon leakage from the therapeutic device, radiation derived from the therapeutic collimator, and scattering of radiation in the patient—the size of the irradiation field, the angle relative to the fetus, the distance from the fetus, and the dose given, are important factors to consider [4, 20, 39]. It has been reported that radiation therapy for oral cancer can safely be performed because of the attenuation effect by using a shielding object and distance [4]. In case 4, since there were two metastatic lung lesions, stereotactic radiotherapy (48 Gy/4 times) was performed at 17 weeks’ gestation; no effect on the fetus was observed. The summary of 11 cases, including the present patient, of radiotherapy for oral cancer during pregnancy is presented in Table 3 [4–6, 8, 11, 22]. Spontaneous abortion was reported in 1 case (9.1%), and the maternal death was reported in 3 cases (27.3%).

**Table 3:** Characteristics of 11 cases of pregnancy

Characteristics		
Age, year (average)	26–38	(30.1)
Age of pregnancy at diagnosis, weeks (average		
	5–28	(16.5)
Age of pregnancy at treatment, weeks (average)		
	7–27	(20.1)
Stage		
II	3	(27.3%)
IV	6	(54.5%)
NA	2	(18.2%)
A dose of radiotherapy, Gy (average)		
	39–66	(54.9)
Fetal outcome		
Delibery	7	(63.6%)
Cesarean	3	(27.3%)
Spontaneous abortion	1	(9.1%)
Maternal outcome		
NED	7	(63.6%)
Died	3	(27.3%)
NA	1	(9.1%)

*NA* not available, *NED* not evidence disease

Systemic Therapy Conditions.

In terms of chemotherapy, the timing of its use is especially important. If administered in the second and third trimesters, after organogenesis has taken place, the incidence of in utero fetal development failure and malformation is said to be not much different from that in observed in the general population. A three-week interval between labor and chemotherapy is recommended to avoid fetal myelosuppression [40]. Given the increased plasma volume and changes in circulatory dynamics that occur during pregnancy, the optimal dose of chemotherapeutic drugs has not been conclusively determined. Although many anticancer drugs undergo placental transfer, fetal blood concentrations of these drugs are reportedly lower than maternal blood concentrations because of placental drug transporter protein expression and adjustment [40]. In case 4, when pulmonary metastases were detected, chemoradiotherapy (with cisplatin and 5-fluorouracil) was recommended. However, the patient and her family refused chemotherapy; hence, only radiotherapy was administered during pregnancy.

## Conclusion

In conclusion, due to social changes in terms of the increasing age of childbearing, the incidence of oral cancers in pregnant women is anticipated to increase. When deciding on a treatment strategy, multiple factors must be considered including patient and family wishes, social background, gestational age, and tumor stage. Moreover, a treatment strategy should be planned in a multidisciplinary fashion.

## Abbreviations

CT: Computed tomography
MRI: Magnetic resonance imaging
PET/CT: Positron emission tomography/ CT
SCC: Squamous cell carcinoma
